# Data-warehousing of protein-protein interactions indicates that pathogens preferentially target hub and bottleneck proteins

**DOI:** 10.3389/fmicb.2013.00051

**Published:** 2013-03-11

**Authors:** Sylvia Schleker, Mirko Trilling

**Affiliations:** ^1^Institute of Complex Systems, Molecular Biophysics (ICS-5), Forschungszentrum Juelich GmbHJuelich, Germany; ^2^Institute for Virology, University Duisburg-Essen, University Hospital EssenEssen, Germany

When pathogens come into contact with their respective host, they immediately encounter the broad diversity of the current cellular proteome which usually consists of several thousand different proteins spanning a concentration range of at least seven orders of magnitude (Beck et al., 2011). Pathogens exploit the host, its metabolism and proteome for their own replication. The resulting expenses for the host provoke the evolution of immune mechanisms. Immune reactions are initiated by receptors which recognize conserved pathogen-associated patterns and signal via highly interconnected networks of co-receptors, adapter proteins, and transcription factors to induce the expression of anti-pathogenic host proteins. Pathogens in turn develop corresponding countermeasures like pathogen-encoded immune antagonists and bacterial effector proteins. These facts exemplify the complexity and richness of the interactions between host- and pathogen-derived proteomes.

A considerable fraction of host proteins directly or indirectly influences the replication of pathogens. Irrespective of whether a protein supports or restricts replication, the functional understanding of this interaction can in principle be utilized for therapeutic interventions: Anti-pathogenic proteins and compounds can be exogenously administered (like e.g., recombinant interferons) whereas proteins which support the replication of a pathogen (e.g., entry receptors) constitute potential drug targets. Therefore, an in-depth knowledge of protein-protein interactions (PPIs) between host and pathogens is highly desirable. In the past, most PPIs have been identified in small scale experiments. Recently, large-scale screens (e.g., yeast-2-hybrid screens) have been used to unravel a broader picture of the global PPI networks.

Durmuş Tekir et al. (2012) developed a novel pathogen-host interaction (PHI) search tool called PHISTO (www.phisto.org) and compared the retrieved PHIs with the network of naive human PPIs which had been interrogated from the databases BioGRID, DIP, IntAct, Mint, and Reactome. The resulting interaction data comprise over 23,000 PPIs derived from 72 different pathogens. The authors assessed the tendency of pathogen-derived proteins to interact with host hub and/or bottleneck proteins. Hub proteins are defined by their above-average number of direct interactions, whereas bottleneck proteins exhibit a high betweenness centrality - meaning that an over-proportional number of connections between two other proteins in the network go through the respective protein. Consistent with previous reports (Calderwood et al., 2007; de Chassey et al., 2008; Dyer et al., 2008, 2010; Wuchty et al., 2010; Zhao et al., 2011), Durmuş Tekir et al. (2012) find that pathogen proteins have the significant tendency to interact with hub and bottleneck proteins of the host. Proteins which are targeted by 2 (or 3) different viral or bacterial pathogens show an increasing centrality within the PPI network of the host. This finding certainly makes sense. During infection, relatively few pathogen-derived proteins face a huge number of host proteins. In many cases, pathogens have significantly smaller coding capacities than their respective host species, for instance, numerous viruses express less than 15 different proteins and several bacteria translocate a limited number of effector proteins into the host cell. Pathogens seemingly solved the discrepancy between the restricted number of own proteins and the huge host proteome by targeting central elements of the human PPI network. A comparison of bacteria and viruses revealed that this trend is even more pronounced in the case of viruses. Potential explanations might reside in the obligatory intracellular replication or the smaller genome size of viruses. Thus, it might be worthwhile to differentiate bacteria into intracellular and extracellular pathogens and to order the pathogens according to their respective coding capacity and/or the number of pathogen effector proteins.

For the majority of organisms the elucidation of interactomes is still in its infancy and countless PPIs remain to be discovered and characterized. Research in this field has to address some important questions. For instance, by far none of the currently available databases is complete. Most databases lack PPIs which have been individually identified and published but not listed in databases. For example, at the time of the analysis, PHISTO contained only five *Salmonella*-human PPIs - the ones automatically retrievable from databases like DIP and IntAct - but misses 38 other known *Salmonella*-human PPIs (Schleker et al., 2012). These numbers highlight the potential of “hidden” information that are not easily accessible in an automated fashion. Gathering all the existing information in one single database is obviously a huge challenge, but would significantly stimulate the field. Furthermore, PPI networks are highly dynamic and critically depend on factors like cell type, pathogen strain, duration of infection, pathogen inoculum, and several other experimental conditions. Therefore, a fair assessment of the reproducibility and quality of results are urgently needed and secondly, the integration of experimental results and PPI data is essential to retrieve meaningful interactomes.

In conclusion, large-scale multi-species comparisons like done by Durmuş Tekir et al. (2012) provide valuable insights into general mechanisms of PPIs between host and pathogen. A lot of effort went into identifying PPIs and comparing interactomes but still numerous questions remain to be answered. For instance, how strong is the preference of pathogen proteins for hub and bottleneck proteins? Is it possible to turn the table and argue that if a given human protein is targeted by several pathogens, but neither has many intraspecies PPIs nor constitute a bottleneck for certain pathways, that we might have missed some of its natural interactions? Maybe it might be a good idea to reassess its interactive behavior to unravel currently unknown interactions? Thereby, knowledge about host-pathogen PPIs would not only allow to uncover the pathogens' virulence strategies but might also aid in identifying host proteins and pathways which are of central importance in other human diseases (Schleker et al., 2013). Continued studies and increasing amounts of precise information will further deepen our understanding of the interplay between pathogens and their respective hosts and hopefully identify some common Achilles' heels.
